# Outcomes of cochlear implantation in children with and without inner ear malformations

**DOI:** 10.12669/pjms.342.14066

**Published:** 2018

**Authors:** Mustafa Celik, Erkan Karatas, Muzaffer Kanlikama

**Affiliations:** 1 Mustafa Celik, Department of Otorhinolaryngology, Harran University Medical Faculty, Sanlıurfa, Turkey; 2 Erkan Karatas, Department of Otorhinolaryngology, Inonu University Medical Faculty, Malatya, Turkey; 3 Muzaffer Kanlikama, Department of Otorhinolaryngology, Gaziantep University Medical Faculty, Gaziantep, Turkey

**Keywords:** Cochlear implantation, Congenital inner ear anomaly, Sensorineural hearing loss, Auditory performance, Speech development

## Abstract

**Objective:**

To evaluate the auditory functions and progress of speech development in children with and without cochlear anomalies who underwent cochlear implantation due to prelingual profound sensorineural hearing loss (SNHL).

**Methods:**

This study was conducted at Gaziantep University Faculty of Medicine Ear-Nose-Throat Department, between October 2006 and December 2007. A total of 69 children (aged 6 to 24 months) diagnosed with profound SNHL were included. Patients were divided into two groups with respect to the presence of inner ear anomalies: Group-1 consisted of 41 children without inner ear anomaly, whereas Group-2 was composed of 28 patients with inner ear anomalies. The auditory performance was assessed using Listening Progress Profile Test (LPPT) and Monosyllabic Trochee Polysyllabic Test (MTP), the subsections of Evaluation of Auditory Responses to Speech (EARS) test battery.

**Results:**

Preoperative LPPT scores were 5 (12%) in both groups. Mean LPPT values after fitting in Group-1 and Group-2 on 1^st^, 3^rd^ and 6^th^ months were 18.5 (44.1%) and 19 (45.6%); 27 (64.2%) and 28 (67.3%); 31 (75%) and 34 (83%), respectively. Postoperatively, MTP scores in Group-1 and Group-2 were 7.5 (62%) and 7.7 (64%) for 3-words set; 10.4 (58%) and 10.6 (59%) for 6-words set; 14.3 (60%) and 14 (59%) for 12-words set, respectively. The rate of stimulation for electrodes was 1345 q/u (quick/unit) in Group-1 and 1310 q/u in Group-2. No statistically significant difference was detected between groups for variables under investigation.

**Conclusion:**

Cochlear implantation is an effective treatment in children with prelingual profound SNHL. Auditory performance and advancement of speech are similar for children with and without inner ear anomalies.

## INTRODUCTION

Hearing loss is an important disability in our country and over the globe. This problem prevents social harmony and leads to the emergence of individual and social problems. For years, vigorous efforts have been made to help these patients to gain their hearing abilities, and to develop speech. Nowadays, cochlear implant system which has been successfully applied all over the world can recover hearing ability of these patients.^1^,^2^ For a successful cochlear implant surgery, anatomical structures of the inner ear and auditory nerve should be intact. Sometimes, despite the presence of the structures of the inner ear, they may not have a typical appearance. Imaging modalities detect malformations of the inner ear structures of the children born with hearing loss.^3^,^4^ Using short and compressed electrodes manufactured for the children with these types of malformations, cochlear implantation can be achieved.

In this study, outcomes of the cochlear implant in pediatric patients with very advanced prelingual sensorineural hearing loss (SNHL) with or without congenital inner ear malformations were measured. Hearing functions and development of speech were comparatively evaluated.

## METHODS

This study took place in, Gaziantep University Faculty of Medicine Ear-Nose-Throat Department, between October 2006 and December 2007. A total of 69 cases with prelingual bilateral profound SNHL or developed within the first two years of life were included in the study. The cases that underwent cochlear implantation surgery at least six months ago were included. The cases were divided to two groups, those without inner ear anomaly (n=41; Group-1) and with inner ear anomaly (n=28; Group-2). The Gaziantep University Local Ethical Committee approved this study (05-2007/ 17).

**Fig.1:**
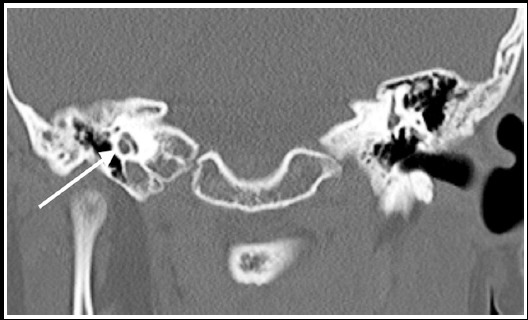
Thin-slice coronal computed tomographic image of a sample case with normal inner ear anatomy obtained before cochlear implantation surgery (Cochlea has 2.5 spirals, vestibule and other structures are natural).

**Fig.2:**
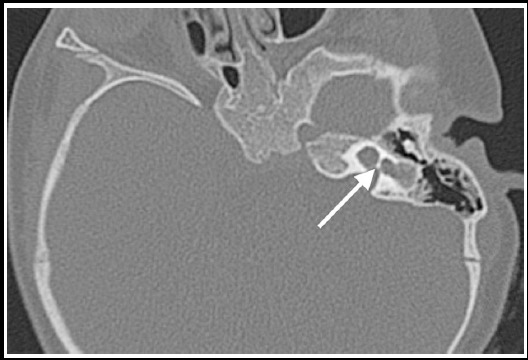
Thin-slice coronal computed tomographic image of a sample case with inner ear malformation obtained before cochlear implantation surgery (Cochlea has not 2.5 spirals and only one cochleo-vestibular cavity is found).

**Fig.3:**
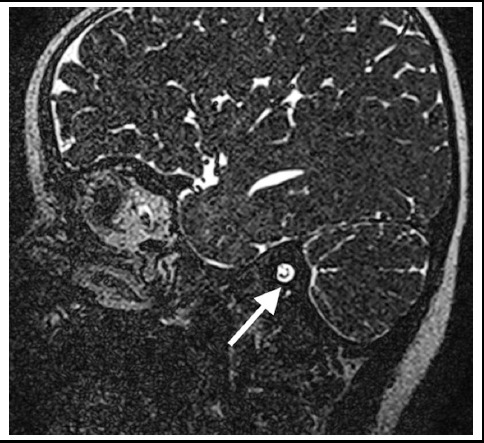
Thin-slice coronal computed tomographic image of a sample case with inner ear malformation obtained before cochlear implantation surgery demonstrating cochlear nerve in the internal acoustic channel included in the 4 nerve combination.

**Fig.4:**
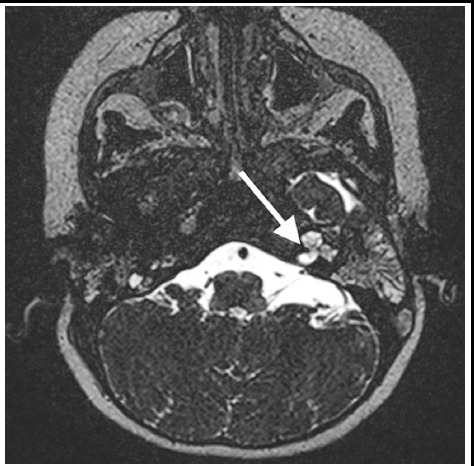
Thin-slice axial coronal computed tomographic image of a sample case with inner ear malformation obtained before cochlear implantation surgery, displays cochlea, and vestibulum in a common cavity containing inner ear fluid.

The cases underwent pure tone/free field audiometry, tympanometry, acoustic reflex measurements, brainstem audiometry (BSA) and auto acoustic emission (OAE) tests before cochlear implantation. All the patients were referred to the departments of pediatric psychiatry and neurology for evaluation. Audiological data of the patients and auditory perception performances were evaluated by an audiologist specialized in this field. Patients who did not benefit from hearing aids, and were prospective cochlear implant candidates, underwent thin-slice axial and coronal computed tomographic (CT) and temporal magnetic resonance imaging (MRI) techniques (Fig.1-4). In this study, the classification developed by Sennaroglu et al. based on data obtained during CT and MRI of 23 patients with inner ear malformations was used.

Cochlear implant surgery was performed using modified minimal postauricular incision, mastoidectomy, posterior tympanotomy and oval window approach under general anesthesia. A 28 mm- long multi-channel Medel Cochlear Implant Device (Medel Pulsar Cl 100, Austria) electrode was implanted. For patients with inner ear malformations 21mm-long relatively thinner Medel Cochlear Implant Device (Medel Pulsar Cl 100, Austria) electrode was used. Electrodes were controlled intraoperative using telemetric measurement, and acoustic stapes reflex was examined. Telemetric measurements were performed using a fitting device (Medeldib II, Austria) and these measurements were recorded using Maestro by Medel fitting program. To assess and record the correct placement implant Stenvers and transorbital petrous skiagram were obtained during early postoperative period.

Auditory performances of the cases were evaluated after cochlear implantation using Evaluation of Auditory Responses to Speech (EARS) test battery. Auditory performances of both groups were compared with subtest groups of EARS test battery including Listening Progress Profile Test (LPPT) and Monosyllabic Trochee Polysyllabic Test (MTP) scores. MTP test was performed for patients whose Listening Progress Profile Test scores were ≥ 30 points. Pre- and postoperative test results of all patients were compared. Besides EARS test results of the patients with or without inner ear malformations who used cochlear implants for nearly equal time periods were also compared. After wound healing all the cases were referred for rehabilitation program for switch on, programming, speech training and progress evaluation.

### Statistical analysis

Velocity values detected in postoperative telemetric measurements, pre-and post-operative EARS test results were statistically compared using Mann-Whitney-U test. p<0.05 values were considered to be significant.

## RESULTS

In this study, Group-1 consisted of 41 (23 males and 18 females) patients with a mean age of 14.24±4.15 months. The Group-2 comprised of 28 (16 males, 12 females) patients with a mean age of 12.64±3.85 months. Cochlear implants were applied for the right (n=37; 54.2%) and left (n=32; 45.7%) ears of the patients. Etiologies of the hearing loss were detected as idiopathic causes (n=30; 47.4%) febrile disease (n=15; 22.03%), familial genetic disease (n=14; 20.03%), difficult labor (n=6; 6.7%) and squeal of meningitis (n=4; 3.3%) (Table-I). Successful outcomes were detected in 68 of 69 patients (98.5%), while in one patient improvement in hearing functions could not be precisely detected because of the presence of auditory neuropathy. In 28 of 69 patients inner ear malformations of various forms were detected including cochlear hypoplasia (n=8), incomplete partition Type-2 (Mondini deformity. n=7), incomplete partition Type-1 (n=5), labyrinthitis ossificans (n=4), common cavity (n=2) and Michel’s aplasia (n=2) (Table-II).

**Table-I:** Etiologies of the hearing loss.

	n	%
Idiopathic causes	30	47.40
Febrile disease	15	22.03
Familial genetic disease	14	20.03
Difficult labor	6	6.7
Squeal of meningitis	4	3.3

Total	69	100

**Table-II:** Patients characteristics and their respective populations.

Patients	(n,%)
Normal anatomy	41(59.4%)
Cochlear hypoplasia	8(11.5%)
Incomplete partition type-2 (Mondini deformity)	7(10.1%)
Incomplete partition type-1	5(7.2%)
Labyrinthitis ossificans	4(5.7%)
Common cavity	2(2.8%)
Michel’s aplasia	2(2.8%)

Total	69(100%)

LPPT scores of the patients with (Group-2) or without cochlear malformations (Group 1) were evaluated during preoperative period and at one, three and six months after fitting. After first fitting median values were as follows as depicted in Table-III. Average preoperative LPPT score of both groups was five points (12%). Average values detected in both groups after the first fitting were as follows: at one. month: Group 1, n=18.5 (44.1%) and Group 2; n=19 (45.6%); at 3. month: Group-1, n=27 (64.2%) and Group 2, n=28 (67.3%); and at 6. month: Group 1, n=31 (75 %), and Group 2, n=34 (83%). Statistically significant difference was not detected between both groups (p>0.05).

**Table-III:** Listening Progress Profile Test (LPPT) results of Group-1 and Group-2 during preoperative period and at 1, 3, and 6 months after fitting.

LPPT Values (Points/ %)	Preoperative	1. month	3. month	6. month
Group-1	5(12%)	18.5(44.1%)	27(64.2%)	31(75%)
Group-2	5(12%)	19.0(45.6%)	28(67.3%)	34(83%)

Pre- and postoperative 6. month-MTP scores of Groups 1 and 2, were evaluated. Preoperative MTP score was 0 percent. Postoperatively, MTP scores in the 3-words-set were 7.5 (62%) in Group-1 and 7.7 (64%) in Group-2; in the 6-words-set MTP scores were 10.4 (58%) in Group-1 and 10.6 (59%) in Group-2; in the 12-word set MTP scores were 14.3 (60%) in Group-1 and 14 (59%) in Group-2 as depicted in Table-IV. Statistically significant difference was not detected between both groups (p>0.05).

**Table-IV:** Preoperative and postoperative 6 months Monosyllabic Trochee Polysyllabic Test (MTP) scores Groups 1 and 2.

MTP Values (Score/ %)	Preoperative	3-word-groups	6-word groups	12-word groups
Group-1	0	7.5(62%)	10.4(58%)	14.3(60%)
Group-2	0	7.7(64%)	10.6(59%)	14.0(59%)

The rate of stimulation for electrodes was 1345 q/u (quick/unit) in Group-1 and 1310 q/u in Group-2. Statistically significant difference was not detected between both groups (p>0.05).

## DISCUSSION

There are nearly 250 million people with hearing loss all over the world.^5^ In Turkey 158.226 patients with hearing loss, less than 18 years of age have been estimated.^6^ In our country, every year nearly 500 babies are born with hearing loss of varying degrees.^7^ The incidence of hearing loss is 1-6/1000 in newborns, and this rate rises to 10:1000 among risky babies. Hearing loss is 20 times higher than that found in screening tests for phenylketonuria performed for every newborn.^5^ Delay in diagnosis leads to important loss of time, that hinders development of speech, mental and social skills. On the other hand, it is also very important for earlier recognition of the individuals with hearing loss, their appropriate rehabilitation, and reintegration into the society with better quality of life.

Hearing loss can cause sensorial deprivation in the communication skills and regression in the development of language skills and learning ability during infancy. For instance, even children with mild degrees (35-40 dB) of hearing loss can miss 50% of the verbal communication in daily life if they don’t use any hearing aid. Therefore, these types of school age children should use a suitable hearing aid within the first six months and continue their education either by using this device or a cochlear implant dependent on the severity of hearing loss.

Etiologies of cases with hearing loss are multifactorial. In a study by Daya et al. on 80 cases with cochlear implants, the idiopathic hearing loss was detected in 62.5% of the patients, while Brookhouser et al. reported the presence of idiopathic hearing loss in 31.5 % of their series consisting of 200 cases.^8^,^9^ However, in our study idiopathic hearing loss was seen in 47.4% of the cases which is closer to the average values of both studies. Karatas et al. reported a higher incidence of familial-genetic history in hearing loss in a region where consanguineous marriages are more prevalent.^10^ In our study, its incidence was found to be 20.03 percent.

During rehabilitation process after cochlear implantation, EARS test battery is used to follow up developmental levels of the patients. Our LPPT and MTP test results are consistent with the literature.^2^,^11^

Literature reviews have shown that speech perception performance and auditory functions in anomalous ears improve at a higher rate during the postoperative period.^12^-^14^ Weber et al. investigated congenital malformations in patients with cochlear implants and evaluated findings and postoperative rehabilitation results of 30 pediatric patients. They concluded the results are extremely encouraging.^13^ Slattery and Luxford, meticulously evaluated postoperative data of 7 pediatric patients with inner ear malformations who had undergone cochlear implantation and concluded the presence of similar characteristics with individuals with normal inner ears but had cochlear implants.^15^ Studies have also revealed that auditory functions of the patients with inner ear anomalies who had undergone cochlear implantation were having competitive results with those without inner ear malformations.^16^-^18^ In our study, we detected high rates of improvement in auditory and speech perception performances of children with or without inner ear malformations following cochlear implantation.

Successful results were obtained in both groups. Our results obtained were generally consistent with the literature.^2^,^11^-^2^,^17^-^18^ Despite many studies examining the improvements in speaking performance and auditory functions separately after cochlear implantation surgery are found in the literature, scarce number of studies have comparatively evaluated both groups in combination. From this perspective, our study is an original investigation. Although our study had limitations as relatively small number of cases were included, and being a single-center study, our results obtained will inspire and encourage the surgeons performing cochlear implantation to conduct similar studies on larger scale.

In conclusion, auditory performance and advancement of speech are similar for children with and without inner ear anomalies. Pediatric cochlear implants can achieve development of hearing and speaking in both groups with similarly higher success rates.

### Authors’ Contribution

**MC:** Conceived, designed and did statistical analysis & editing of manuscript.

**MC and EK:** Did data collection and manuscript writing.

**MC and MK:** Takes the responsibility and is accountable for all aspects of the work in ensuring that questions related to the accuracy or integrity of any part of the work are appropriately investigated and resolved.
